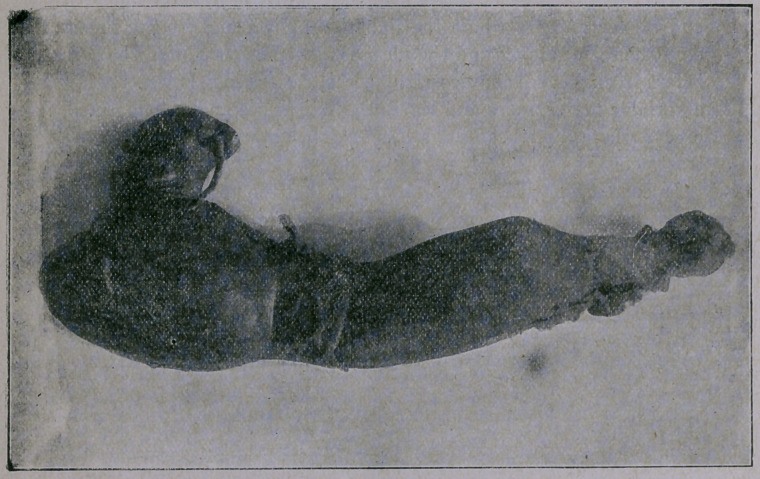# Intestinal Obstruction from Meckel’s Diverticulum*Paper read at meeting of the Richmond Academy of Medicine and Surgery, January 12, 1904.

**Published:** 1904-02

**Authors:** Stuart M’Guire

**Affiliations:** Surgeon in Charge St. Luke’s Hospital, Richmond, Va.


For Texas Medical Journal.

### Intestinal Obstruction from Meckel's Diverticulum.*

BY STUART M'GUIRE, M. D., SURGEON IN CHARGE ST. LUKE'S HOSPITAL, RICHMOND, VA.

Whether admitted or not there is undoubtedly a general belief among surgeons that cases occur in groups, and I confess the superstition has been brought home to me by three cases of intestinal obstruction, due to Meckel's diverticulum, that have recently occurred in my practice. The symptoms, pathologic conditions and final results were so similar in all that it is unnecessary to give a separate history of each. All were men between twenty and thirty years of age; all were taken with sudden abdominal pain, followed by obstruction, distension and peritonitis; all were brought to the hospital practically moribund from sepsis; all were diagnosticated as fulminating appendicitis; all were operated upon, and all died.

In each case where the abdomen was opened, there was the escape

*Paper read at meeting of the Richmond Academy of Medicine and Surgery, January 12, 1904.

of a quart or more of bloody serum; in each the bowels were inflamed and distended with gas, and in each a gangrenous diverticulum was found, originating from the ileum, extending upward and inward to be attached by its tip to the mesentery, and having beneath it an incarcerated coil of small intestine. The specimen I exhibit was removed from the last case. It was seven inches in length, one inch in its smallest diameter and is expanded at its tip into a sacculated cavity.

The rapidity with which a strangulated diverticulum kills, and the necessity of surgical intervention even more prompt than in appendicitis, has led me to study the available literature on the subject and to report the rather unsatisfactory result.

In early fetal development the intestinal canal communicates with the vitelline sac by means of the vitelline or amphalo mesenteric duct. This duct begins at the lower end of the ileum and passes through the abdominal wall at the site of the future umbilicus. It usually becomes obliterated at the end of the sixth week. If it does not undergo atrophy a diverticulum results shaped like a glove finger, with its base opening into the bowel and its tip either floating free in the abdominal cavity or attached by a fibrous cord to the umbilicus. Meckel's diverticulum varies in length from one to ten inches, and in diameter from a scarcely permeable tube to a protrusion the caliber of the small intestine. It is usually cylindrical in shape, but may be sacculated or expanded into cavities. The distal extremity may be smooth and tapering or it may be rough and bulbous. It is usually located about three feet above the ileo cecal valve on the convex side of the intestine opposite the insertion of its mesentery.

If free the distal end may become adherent to any place within the abdominal cavity its length permits it to reach. Its most frequent point of attachment is the mesentery, although a case is reported where it was fastened to the bladder.

When Meckel's diverticulum is connected with the umbilicus by a fibrous cord it may cause intestinal obstruction by a loop of the bowel becoming twisted around it. When it floats free in the abdominal cavity it may cause obstruction either by encircling a bowel and becoming mechanically locked by its club shaped extremity or by the free end becoming attached to a fixed point by inflammatory adhesions and a loop of intestines being caught beneath it.

Meckel's diverticulum is said to exist in about 2 per cent. of all bodies examined. I have accidentally observed its presence several times while operating for other abdominal troubles. As the victim of the abnormality usually goes through life unconscious of its existence, and as only a small per cent. have abdominal obstruction, the number of cases reported is not large.

The symptoms due to strangulation by the diverticulum are sudden in onset. Pain is severe and persistent and referred chiefly to the region of the umbilicus. Vomiting appears early and may become stercoraceous; tenesmus and discharge of blood from the rectum are absent; constipation is, as a rule, absolute; the abdominal wall is not rigid, but later becomes tense from distension; fever and the attending symptoms of sepsis begin with the development of peritonitis, and sometimes there is tenderness or a perceptible swelling near the umbilicus.

All writers admit that it is impossible to make a positive diagnosis in a case of intestinal obstruction due to the diverticulum, or to differentiate it from intestinal paresis due to peritonitis of appendicular origin, hence the importance of early operative intervention in doubtful cases.

Ochsner's method of treatment of peritonitis, while valuable in appendicitis, would prove uniformly fatal in mechanical obstruction. The abdomen should be opened in the middle line and the lower right quadrant first examined. If there is a large quantity of bloody serum free from the admixture of pus a strangulated diverticulum will most likely be found. As soon as it is located the tip should be separated from the tissue to which it is adherent and the obstruction relieved. The patency of the bowel should then be demonstrated and its walls carefully examined to see if they are damaged sufficiently to necessitate resection. Finally the diverticulum should be removed. If it is small it may be tied and amputated like an appendix, the stump being buried or covered with peritoneum. If it is large it would be unsafe to trust to a ligature, as it might cut through and cause death from peritonitis at a time when the patient was regarded as out of danger. If the size of the diverticulum approaches that of the ileum from which it originates it should be amputated and the opening closed with the same care and by the same methods as an intestinal wound of the same size from other causes.

## Figures and Tables

**Figure f1:**